# Supplementary figures and images for: Evidence of potential impacts of a nutrition-sensitive agroecology program in Andhra Pradesh, India, on dietary diversity, nutritional status, and child development
Source: PLoS One. 2024 May 13;19(5):e0286356. doi: 10.1371/journal.pone.0286356 (PMC11090352; doi:10.1371/journal.pone.0286356)

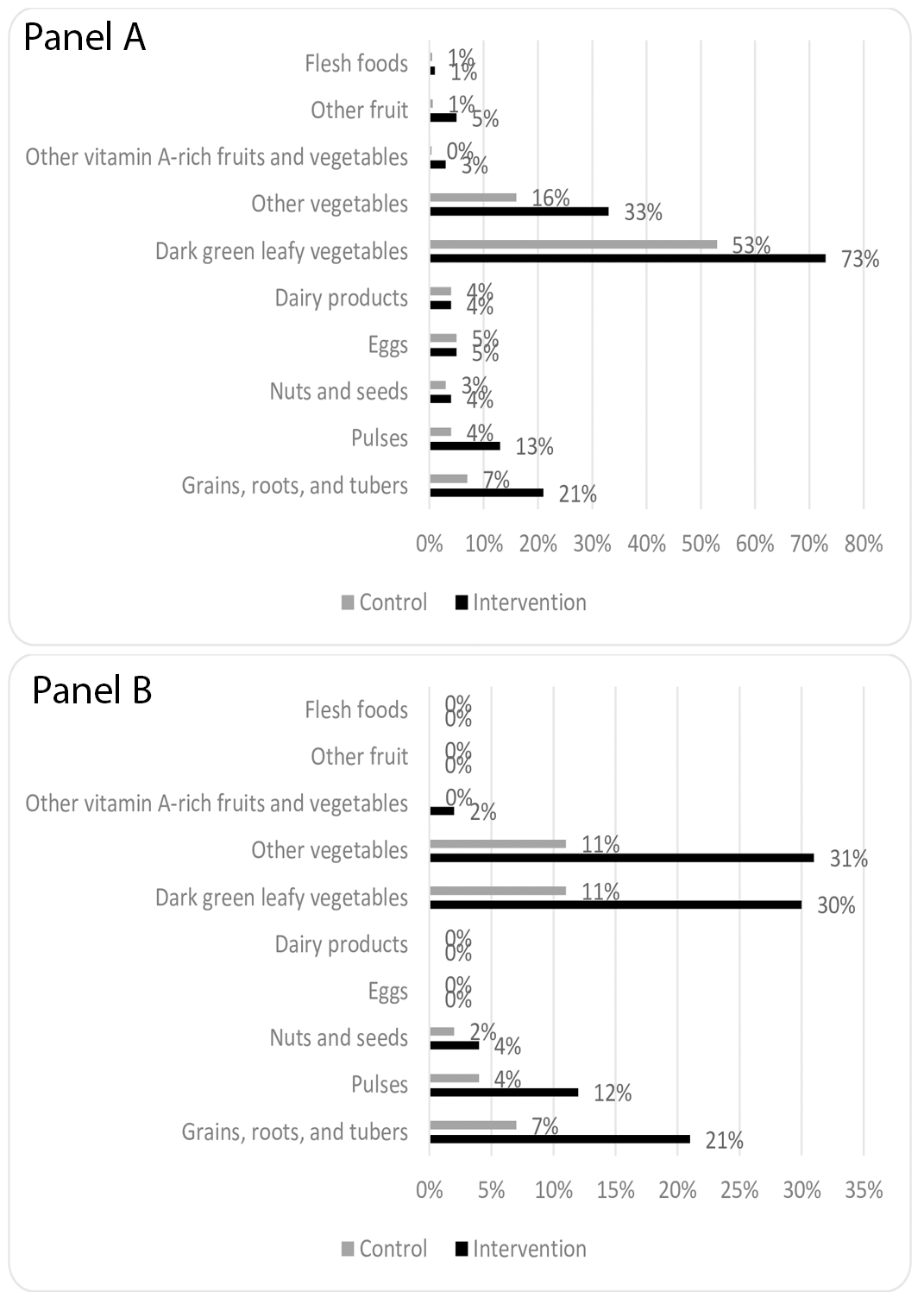

Supplement: S1 Fig — Naturally farmed food groups consumed by women (Panel A) and men (Panel B) in intervention versus control villages. (TIF) [file pone.0286356.s001.tif]

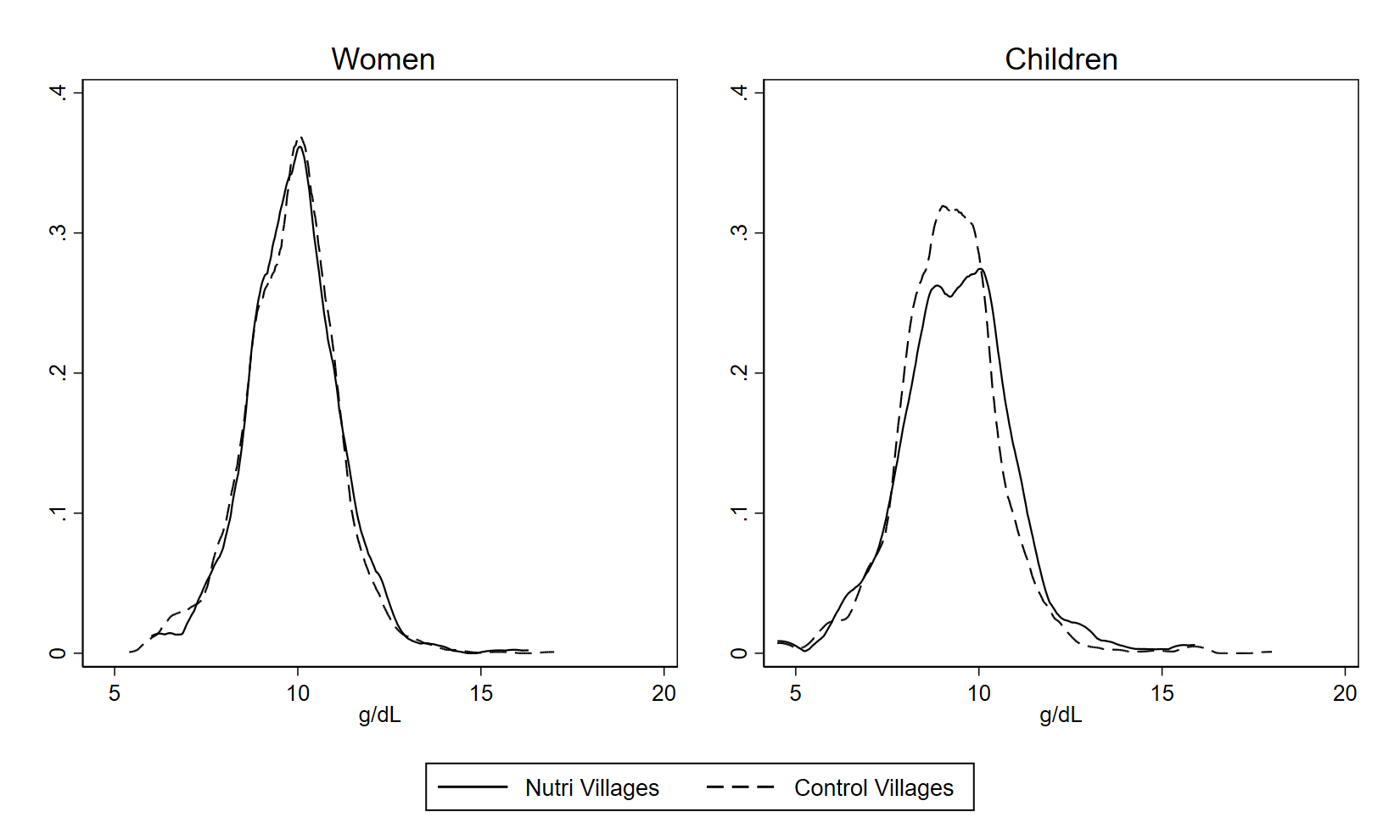

Supplement: S2 Fig — Distribution of haemoglobin in women (Panel A) and children (Panel B). (TIF) [file pone.0286356.s002.tif]
