# Supplementary material for: Evidence of potential impacts of a nutrition-sensitive agroecology program in Andhra Pradesh, India, on dietary diversity, nutritional status, and child development
Source: PLoS One. 2024 May 13;19(5):e0286356. doi: 10.1371/journal.pone.0286356 (PMC11090352; doi:10.1371/journal.pone.0286356)
Supplement: S1 Table — (DOCX) [file pone.0286356.s003.docx]

## Supplementary Table T1: Dietary Diversity Scores among men and women

### For mothers:

|  |  | **Unadjusted** | | | **Adjusted*** | | |  |
| --- | --- | --- | --- | --- | --- | --- | --- | --- |
|  | **Full sample**  **N (%)** | **Intervention villages**  **N (%)** | **Control villages**  **N (%)** | **p-Value** | **Intervention villages**  **N (%)** | **Control villages**  **N (%)** | **p-Value** | |
| Grains, roots, and tubers | 3510 (99.97) | 1120 (99.91) | 2390 (100) | 0.14 | 1120 (99.91) | 2390 (100) | 0.10 | |
| Pulses | 2497 (71.12) | 854 (76.18) | 1643 (68.74) | < 0.01 | 854 (76.18) | 1643 (68.74) | < 0.01 | |
| Nuts and seeds | 2083 (59.33) | 717 (63.96) | 1366 (57.15) | < 0.01 | 717 (63.96) | 1366 (57.15) | < 0.01 | |
| Eggs | 1654 (47.11) | 654 (58.34) | 1000 (41.84) | < 0.01 | 654 (58.34) | 1000 (41.84) | < 0.01 | |
| Dairy products | 3032 (86.36) | 1019 (90.9) | 2013 (84.23) | < 0.01 | 1019 (90.9) | 2013 (84.23) | < 0.01 | |
| Dark green leafy vegetables | 1530 (43.58) | 599 (53.43) | 931 (38.95) | < 0.01 | 599 (53.43) | 931 (38.95) | < 0.01 | |
| Other vegetables | 3348 (95.36) | 1052 (93.84) | 2296 (96.07) | < 0.01 | 1052 (93.84) | 2296 (96.07) | 0.01 | |
| Other vitamin A-rich fruits and vegetables | 563 (16.04) | 269 (24) | 294 (12.3) | < 0.01 | 269 (24) | 294 (12.3) | < 0.01 | |
| Other fruit | 2144 (61.07) | 752 (67.08) | 1392 (58.24) | < 0.01 | 752 (67.08) | 1392 (58.24) | < 0.01 | |
| Flesh foods | 836 (23.81) | 282 (25.16) | 554 (23.18) | 0.20 | 282 (25.16) | 554 (23.18) | 0.86 | |
| Dietary diversity score (0-10) | 6.04±1.63 | 6.53±1.62 | 5.81±1.58 | < 0.01 | 6.53±1.62 | 5.81±1.58 | < 0.01 | |
| Met minimum dietary diversity (DDS>=5) | 2864 (81.57) | 973 (86.8) | 1891 (79.12) | < 0.01 | 973 (86.8) | 1891 (79.12) | < 0.01 | |
| *Adjusted for tribal vs non-tribal village. | | | | | | | |  |

### For men:

|  |  | **Unadjusted** | | | **Adjusted*** | | | |  |
| --- | --- | --- | --- | --- | --- | --- | --- | --- | --- |
|  | **Full sample**  **N (%)** | **Intervention villages**  **N (%)** | **Control villages**  **N (%)** | **p-Value** | **Intervention villages**  **N (%)** | **Control villages**  **N (%)** | **p-Value** | |  |
| Grains, roots, and tubers | 3494 (99.52) | 1114 (99.38) | 2380 (99.58) | 0.41 | 1114 (99.38) | 2380 (99.58) | | 0.41 | |
| Pulses | 2406 (68.53) | 825 (73.6) | 1581 (66.15) | < 0.01 | 825 (73.6) | 1581 (66.15) | | < 0.01 | |
| Nuts and seeds | 1939 (55.23) | 652 (58.16) | 1287 (53.85) | 0.02 | 652 (58.16) | 1287 (53.85) | | < 0.01 | |
| Eggs | 1206 (34.35) | 524 (46.74) | 682 (28.54) | < 0.01 | 524 (46.74) | 682 (28.54) | | < 0.01 | |
| Dairy products | 2830 (80.6) | 985 (87.87) | 1845 (77.2) | < 0.01 | 985 (87.87) | 1845 (77.2) | | < 0.01 | |
| Dark green leafy vegetables | 1433 (40.81) | 564 (50.31) | 869 (36.36) | < 0.01 | 564 (50.31) | 869 (36.36) | | < 0.01 | |
| Other vegetables | 3327 (94.76) | 1045 (93.22) | 2282 (95.48) | 0.01 | 1045 (93.22) | 2282 (95.48) | | 0.03 | |
| Other vitamin A-rich fruits and vegetables | 483 (13.76) | 236 (21.05) | 247 (10.33) | < 0.01 | 236 (21.05) | 247 (10.33) | | < 0.01 | |
| Other fruit | 1811 (51.58) | 671 (59.86) | 1140 (47.7) | < 0.01 | 671 (59.86) | 1140 (47.7) | | < 0.01 | |
| Flesh foods | 844 (24.04) | 285 (25.42) | 559 (23.39) | 0.19 | 285 (25.42) | 559 (23.39) | | 0.72 | |
| Dietary diversity score (0-10) | 5.63±1.66 | 6.16±1.65 | 5.39±1.61 | < 0.01 | 6.16±1.65 | 5.39±1.61 | | < 0.01 | |
| Met minimum dietary diversity (DDS>=5) | 2669 (76.02) | 934 (83.32) | 1735 (72.59) | < 0.01 | 934 (83.32) | 1735 (72.59) | | < 0.01 | |
| *Adjusted for tribal vs non-tribal village. | | | | | | | | |  |
