# Supplementary material for: Evidence of potential impacts of a nutrition-sensitive agroecology program in Andhra Pradesh, India, on dietary diversity, nutritional status, and child development
Source: PLoS One. 2024 May 13;19(5):e0286356. doi: 10.1371/journal.pone.0286356 (PMC11090352; doi:10.1371/journal.pone.0286356)
Supplement: S2 Table — (DOCX) [file pone.0286356.s004.docx]

## Supplementary Table T2: Dietary Diversity Scores among Children <2 and ≥2 years age

### For Children 6-23 months age:

|  |  | **Unadjusted** | | | **Adjusted*** | | | |  |
| --- | --- | --- | --- | --- | --- | --- | --- | --- | --- |
|  | **Full sample**  **N (%)** | **Intervention villages**  **N (%)** | **Control villages**  **N (%)** | **p-Value** | **Intervention villages**  **N (%)** | **Control villages**  **N (%)** | **p-Value** | |  |
| Grains, roots, and tubers | 1410 (69.87) | 474 (69.1) | 936 (70.27) | 0.59 | 474 (69.1) | 936 (70.27) | | 0.20 | |
| Pulses, nuts, and seeds | 1232 (61.14) | 423 (61.66) | 809 (60.87) | 0.73 | 423 (61.66) | 809 (60.87) | | 0.04 | |
| Dairy products | 647 (31.98) | 216 (31.44) | 431 (32.26) | 0.71 | 216 (31.44) | 431 (32.26) | | 0.04 | |
| Flesh foods | 78 (3.87) | 29 (4.23) | 49 (3.68) | 0.54 | 29 (4.23) | 49 (3.68) | | 0.93 | |
| Eggs | 762 (37.67) | 305 (44.4) | 457 (34.21) | < 0.01 | 305 (44.4) | 457 (34.21) | | 0.01 | |
| Vit- A rich fruits and vegetables | 734 (36.39) | 313 (45.69) | 421 (31.61) | < 0.01 | 313 (45.69) | 421 (31.61) | | < 0.01 | |
| Other fruits and vegetables | 837 (41.39) | 294 (42.86) | 543 (40.64) | 0.34 | 294 (42.86) | 543 (40.64) | | < 0.01 | |
| Dietary diversity score (0-8) | 3.55±1.54 | 3.71±1.48 | 3.47±1.56 | < 0.01 | 3.71±1.48 | 3.47±1.56 | | < 0.01 | |
| Met minimum dietary diversity (DDS>=5) | 542 (26.79) | 207 (30.13) | 335 (25.07) | 0.02 | 207 (30.13) | 335 (25.07) | | < 0.01 | |
| *Adjusted for tribal vs non-tribal village and child age. | | | | | | | | |  |

### For Children ≥2 years age:

|  |  | **Unadjusted** | | | **Adjusted*** | | | |  |
| --- | --- | --- | --- | --- | --- | --- | --- | --- | --- |
|  | **Full sample**  **N (%)** | **Intervention villages**  **N (%)** | **Control villages**  **N (%)** | **p-Value** | **Intervention villages**  **N (%)** | **Control villages**  **N (%)** | **p-Value** | |  |
| Grains, roots, and tubers | 2164 (99.95) | 763 (99.87) | 1401 (100) | 0.18 | 763 (99.87) | 1401 (100) | | 0.11 | |
| Pulses | 1740 (80.37) | 603 (78.93) | 1137 (81.16) | 0.21 | 603 (78.93) | 1137 (81.16) | | 0.78 | |
| Nuts and seeds | 1153 (53.26) | 434 (56.81) | 719 (51.32) | 0.01 | 434 (56.81) | 719 (51.32) | | < 0.01 | |
| Eggs | 1526 (70.48) | 588 (76.96) | 938 (66.95) | < 0.01 | 588 (76.96) | 938 (66.95) | | < 0.01 | |
| Dairy products | 1829 (84.48) | 681 (89.14) | 1148 (81.94) | < 0.01 | 681 (89.14) | 1148 (81.94) | | < 0.01 | |
| Dark green leafy vegetables | 996 (46) | 434 (56.81) | 562 (40.11) | < 0.01 | 434 (56.81) | 562 (40.11) | | < 0.01 | |
| Other vegetables | 2023 (93.44) | 685 (89.66) | 1338 (95.5) | < 0.01 | 685 (89.66) | 1338 (95.5) | | < 0.01 | |
| Other vitamin A-rich fruits and vegetables | 319 (14.73) | 165 (21.6) | 154 (10.99) | < 0.01 | 165 (21.6) | 154 (10.99) | | < 0.01 | |
| Other fruit | 1312 (60.6) | 523 (68.46) | 789 (56.32) | < 0.01 | 523 (68.46) | 789 (56.32) | | < 0.01 | |
| Flesh foods | 393 (18.15) | 158 (20.68) | 235 (16.77) | 0.02 | 158 (20.68) | 235 (16.77) | | 0.50 | |
| Dietary diversity score (0-10) | 6.21±1.61 | 6.59±1.69 | 6.01±1.53 | < 0.01 | 6.59±1.69 | 6.01±1.53 | | < 0.01 | |
| Met minimum dietary diversity (DDS>=5) | 1832 (84.62) | 660 (86.39) | 1172 (83.65) | 0.09 | 660 (86.39) | 1172 (83.65) | | 0.06 | |
| *Adjusted for tribal vs non-tribal village and child age. | | | | | | | | |  |
