# Supplementary material for: Evidence of potential impacts of a nutrition-sensitive agroecology program in Andhra Pradesh, India, on dietary diversity, nutritional status, and child development
Source: PLoS One. 2024 May 13;19(5):e0286356. doi: 10.1371/journal.pone.0286356 (PMC11090352; doi:10.1371/journal.pone.0286356)
Supplement: S3 Table — (DOCX) [file pone.0286356.s005.docx]

## Supplementary Table T3: Anthropometry in women and children

For women:

|  |  | **Unadjusted** | | | **Adjusted*** | | |
| --- | --- | --- | --- | --- | --- | --- | --- |
|  | **Full sample**  **Mean ± SD** | **Intervention villages Mean ± SD** | **Control villages Mean ± SD** | **p-Value** | **Intervention villages Mean ± SD** | **Control villages Mean ± SD** | **p-Value** |
| Mother's BMI (kg/m2) | 22.66±3.52 | 22.38±3.65 | 22.77±3.46 | 0.02 | 22.38±3.65 | 22.77±3.46 | 0.11 |
| Mother is underweight (<18.5) | 196 (9.67) | 68 (12.23) | 128 (8.7) | 0.02 | 68 (12.23) | 128 (8.7) | 0.10 |
| Mother is normal weight (18.5-24.9) | 1402 (69.17) | 399 (71.76) | 1003 (68.18) | 0.12 | 399 (71.76) | 1003 (68.18) | 0.05 |
| Mother is overweight (25-29.9) | 359 (17.71) | 65 (11.69) | 294 (19.99) | <0.01 | 65 (11.69) | 294 (19.99) | <0.01 |
| Mother is obese (>30) | 70 (3.45) | 24 (4.32) | 46 (3.13) | 0.19 | 24 (4.32) | 46 (3.13) | 0.07 |
| *Adjusted for tribal vs non-tribal village, digital vs analogue scale, and age. | | | | | | | |

In children:

|  |  | **Unadjusted** | | | **Adjusted*** | | |
| --- | --- | --- | --- | --- | --- | --- | --- |
|  | **Full sample**  **N (%)** | **Intervention villages**  **N (%)** | **Control villages**  **N (%)** | **p-Value** | **Intervention villages**  **N (%)** | **Control villages**  **N (%)** | **p-Value** |
| Stunted | 625 (24.4) | 198 (24.15) | 427 (24.53) | 0.83 | 198 (24.15) | 427 (24.53) | 0.38 |
| Wasted | 571 (22.78) | 213 (26.33) | 358 (21.08) | <0.01 | 213 (26.33) | 358 (21.08) | <0.01 |
| Severely wasted | 268 (10.69) | 109 (13.47) | 159 (9.36) | <0.01 | 109 (13.47) | 159 (9.36) | <0.01 |
| Overweight | 130 (5.19) | 45 (5.56) | 85 (5.01) | 0.56 | 45 (5.56) | 85 (5.01) | 0.93 |
| Underweight | 640 (24.47) | 237 (27.75) | 403 (22.88) | 0.01 | 237 (27.75) | 403 (22.88) | 0.01 |
| *Adjusted for tribal vs non-tribal village, digital vs analogue scale, and age. | | | | | | | |
