# Supplementary material for: Evidence of potential impacts of a nutrition-sensitive agroecology program in Andhra Pradesh, India, on dietary diversity, nutritional status, and child development
Source: PLoS One. 2024 May 13;19(5):e0286356. doi: 10.1371/journal.pone.0286356 (PMC11090352; doi:10.1371/journal.pone.0286356)
Supplement: S4 Table — (DOCX) [file pone.0286356.s006.docx]

## Supplementary Table T4: Anaemia in women and children

In women

|  |  | **Unadjusted** | | | **Adjusted*** | | |
| --- | --- | --- | --- | --- | --- | --- | --- |
|  | **Full sample**  **N (%)** | **Intervention villages**  **N (%)** | **Control villages**  **N (%)** | **p-Value** | **Intervention villages**  **N (%)** | **Control villages**  **N (%)** | **p-Value** |
| Severe anaemia (Hb <7 g/dL) | 43 (2.12) | 7 (1.23) | 36 (2.47) | 0.08 | 7 (1.23) | 36 (2.47) | 0.08 |
| Moderate anaemia (7 g/dL <= Hb <10 g/dL) | 1012 (49.9) | 290 (50.88) | 722 (49.52) | 0.58 | 290 (50.88) | 722 (49.52) | 0.98 |
| Mild anaemia (10 gd/L <= Hb <11 g/dL) | 650 (32.05) | 169 (29.65) | 481 (32.99) | 0.15 | 169 (29.65) | 481 (32.99) | 0.34 |
| No anaemia (Hb >=11 g/dL) | 323 (15.93) | 104 (18.25) | 219 (15.02) | 0.07 | 104 (18.25) | 219 (15.02) | 0.06 |
| *Adjusted for tribal vs non-tribal village and age. | | | | | | | |

In children

|  |  | **Unadjusted** | | | **Adjusted*** | | |
| --- | --- | --- | --- | --- | --- | --- | --- |
|  | **Full sample**  **N (%)** | **Intervention villages**  **N (%)** | **Control villages**  **N (%)** | **p-Value** | **Intervention villages**  **N (%)** | **Control villages**  **N (%)** | **p-Value** |
| Severe anaemia (Hb <7 g/dL) | 101 (5) | 40 (5.75) | 61 (4.61) | 0.27 | 40 (5.75) | 61 (4.61) | 0.45 |
| Moderate anaemia (7 g/dL <= Hb <10 g/dL) | 1326 (65.71) | 408 (58.62) | 918 (69.44) | <0.01 | 408 (58.62) | 918 (69.44) | <0.01 |
| Mild anaemia (10 gd/L <= Hb <11 g/dL) | 397 (19.67) | 161 (23.13) | 236 (17.85) | <0.01 | 161 (23.13) | 236 (17.85) | <0.01 |
| No anaemia (Hb >=11 g/dL) | 194 (9.61) | 87 (12.5) | 107 (8.09) | <0.01 | 87 (12.5) | 107 (8.09) | <0.01 |
| *Adjusted for tribal vs non-tribal village and age. | | | | | | | |
