# Supplementary material for: Evidence of potential impacts of a nutrition-sensitive agroecology program in Andhra Pradesh, India, on dietary diversity, nutritional status, and child development
Source: PLoS One. 2024 May 13;19(5):e0286356. doi: 10.1371/journal.pone.0286356 (PMC11090352; doi:10.1371/journal.pone.0286356)
Supplement: S5 Table — (DOCX) [file pone.0286356.s007.docx]

## Supplementary Table T5: Child development scores in children <3 years old by age group

|  |  | **Unadjusted** | | | **Adjusted*** | | |
| --- | --- | --- | --- | --- | --- | --- | --- |
|  | **Full sample**  **Mean ± SD** | **Intervention villages Mean ± SD** | **Control villages Mean ± SD** | **p-Value** | **Intervention villages Mean ± SD** | **Control villages Mean ± SD** | **p-Value** |
| *Child age: 0-5 months* |  |  |  |  |  |  |  |
| N | 650 | 177 | 473 |  | 177 | 473 |  |
| Z-score | -0.45±1.13 | -0.68±1.11 | -0.37±1.12 | < 0.01 | -0.68±1.11 | -0.37±1.12 | < 0.01 |
| *Child age: 6-11 months* |  |  |  |  |  |  |  |
| N | 662 | 221 | 441 |  | 221 | 441 |  |
| Z-score | -0.4±1.23 | -0.35±1.38 | -0.42±1.15 | 0.46 | -0.35±1.38 | -0.42±1.15 | 0.68 |
| *Child age: 12-17 months* |  |  |  |  |  |  |  |
| N | 646 | 235 | 411 |  | 235 | 411 |  |
| Z-score | -0.19±1.06 | -0.1±1.14 | -0.24±1.01 | 0.12 | -0.1±1.14 | -0.24±1.01 | 0.92 |
| *Child age: 18-23 months* |  |  |  |  |  |  |  |
| N | 538 | 185 | 353 |  | 185 | 353 |  |
| Z-score | 0.31±1.09 | 0.46±1.13 | 0.24±1.06 | 0.02 | 0.46±1.13 | 0.24±1.06 | 0.25 |
| *Child age: 24-29 months* |  |  |  |  |  |  |  |
| N | 239 | 82 | 157 |  | 82 | 157 |  |
| Z-score | -0.4±0.79 | -0.14±0.8 | -0.53±0.76 | < 0.01 | -0.14±0.8 | -0.53±0.76 | < 0.01 |
| *Child age: 30-35 months* |  |  |  |  |  |  |  |
| N | 175 | 87 | 88 |  | 87 | 88 |  |
| Z-score | -0.64±0.84 | -0.38±0.76 | -0.91±0.84 | < 0.01 | -0.38±0.76 | -0.91±0.84 | < 0.01 |
| *Child age: 0-35 months* |  |  |  |  |  |  |  |
| *Adjusted for tribal vs non-tribal village. | | | | | | | |
